# Changes in metabolism-related RNA expression in circulating white blood cells of aged individual with physical frailty

**DOI:** 10.18632/aging.206256

**Published:** 2025-05-28

**Authors:** Yuka Okinaka, Yoshihito Suda, Tomoyuki Matsumoto, Ryosuke Kuroda, Yoshiyuki Shinagawa, Sheraz Gul, Carsten Claussen, Ikuko Matsui, Yutaka Matsui, Akihiko Taguchi

**Affiliations:** 1 Department of Regenerative Medicine Research, Foundation for Biomedical Research and Innovation at Kobe, Kobe, Hyogo 650-0047, Japan; 2 Department of Orthopedic Surgery, Kobe University Graduate School of Medicine, Kobe, Hyogo 650-0019, Japan; 3 Global Planning Group, Kaneka corporation, Osaka-shi, Osaka 530-8288, Japan; 4 Fraunhofer Institute for Translational Medicine and Pharmacology ITMP, Hamburg 22525, Germany; 5 Fraunhofer Cluster of Excellence for Immune-Mediated Diseases CIMD, Hamburg 22525, Germany; 6 Matsui Dietary and Dementia Clinic, Akashi-shi, Hyogo 673-0891, Japan

**Keywords:** frailty, circulating leukocyte, quantitative PCR, gap junction, white blood cells

## Abstract

Background: Physical frailty is an age-related clinical condition associated with deterioration of physiological capacity. In aged mice, increased RNA expression of metabolism-related genes in circulating white blood cells (WBC) correlates with impaired physical function.

Methods: Twenty elderly volunteers were enrolled in this exploratory analysis and the possible link between RNA expression of metabolism-related genes in WBC and impairment of their physical function was investigated (jRCT1050210166. Feb. 02, 2022). In mechanism of action studies, cellular interactions between WBC and cells in muscle tissue were investigated in mice.

Results: RNA expression of metabolism-related genes, such as glucose transporter 1 (Glut1), Glut3, AMP-activated protein kinase A, and prolyl hydroxylase 3, was significantly increased in aged individuals with swallowing dysfunction and masticatory disturbance compared to those without these symptoms. The level of metabolism-related RNA expression significantly decreased with walking habits compared to no walking habits. Studies in mice have revealed a direct link between circulating WBC and endothelium/satellite cells via gap junction.

Conclusions: Our results indicate that the level of metabolism-related RNA expression in WBC can serve as a marker of impaired physical function in the elderly and that circulating WBC may have a previously unknown physiological role in maintaining physical function.

## INTRODUCTION

Physical frailty is an age-related clinical condition associated with deterioration in physiological aptitude, resulting in increased susceptibility to stressors [1]. To systematically assess physical frailty in Japan, a national screening program involving elderly individuals was adopted using a new health assessment questionnaire [2]. Complementary work in aged mice has shown increased expressions of metabolism-related RNA, such as glucose transporter (Glut), monocarboxylate transporter (MCT), prolyl hydroxylase 3 (PHD3), and pyruvate dehydrogenase kinase 1 (PDK1), in circulating white blood cells (WBC) are significantly correlated with impaired motor and cognitive functions [3]. WBC have also been shown to transfer water-soluble molecules to the cerebral endothelium via gap junction in mice [4], and *in vitro* analysis revealed that cell-cell interactions between WBC and endothelial cells via gap junction reduce the expression of metabolism-related RNA in WBC [3]. Based on these findings, we had hypothesized that gap junction-mediated cell-cell interaction between WBC and the endothelium activates the endothelium with decreased RNA transcription of metabolism-related genes in WBC, in contrast to impaired cell-cell interaction resulting in no decrease in RNA transcription of metabolism-related genes in WBC with non-activated endothelium in mice [3].

Gap junction is specialized intercellular connections composed of connexins that allow the movement of small water-soluble molecules, including most metabolites, along their concentration gradients [5]. The intracellular concentrations of metabolites vary significantly between cell types, and the concentrations of most glycolytic substrates in WBC and hematopoietic stem cells are higher than those in endothelial cells [6]. Connexins are widely distributed and expressed in almost all tissues, except red blood cells, differentiated skeletal muscle, and mature sperm cells [7]. Gap junction has a significant impact on the metabolic status of connected cells [8] and the major mechanism of action of hematopoietic stem cell therapy aiming for angiogenesis is gap junction mediated small molecules transfer resulting in activation of the metabolic status of endothelial cells though activation of Hypoxia inducible factor 1α (Hif1α) [9].

Based on findings from studies on aged mice and *in vitro* studies [3], we enrolled 20 aged individuals, measured the level of metabolism-related RNA expression in WBC, and assessed the possible link between the level of metabolism-related RNA in WBC and each frailty-based health assessment questionnaire.

## RESULTS

### Changes in WBC RNA expression profiles in elderly with oral and physical function

**Figure 1:**
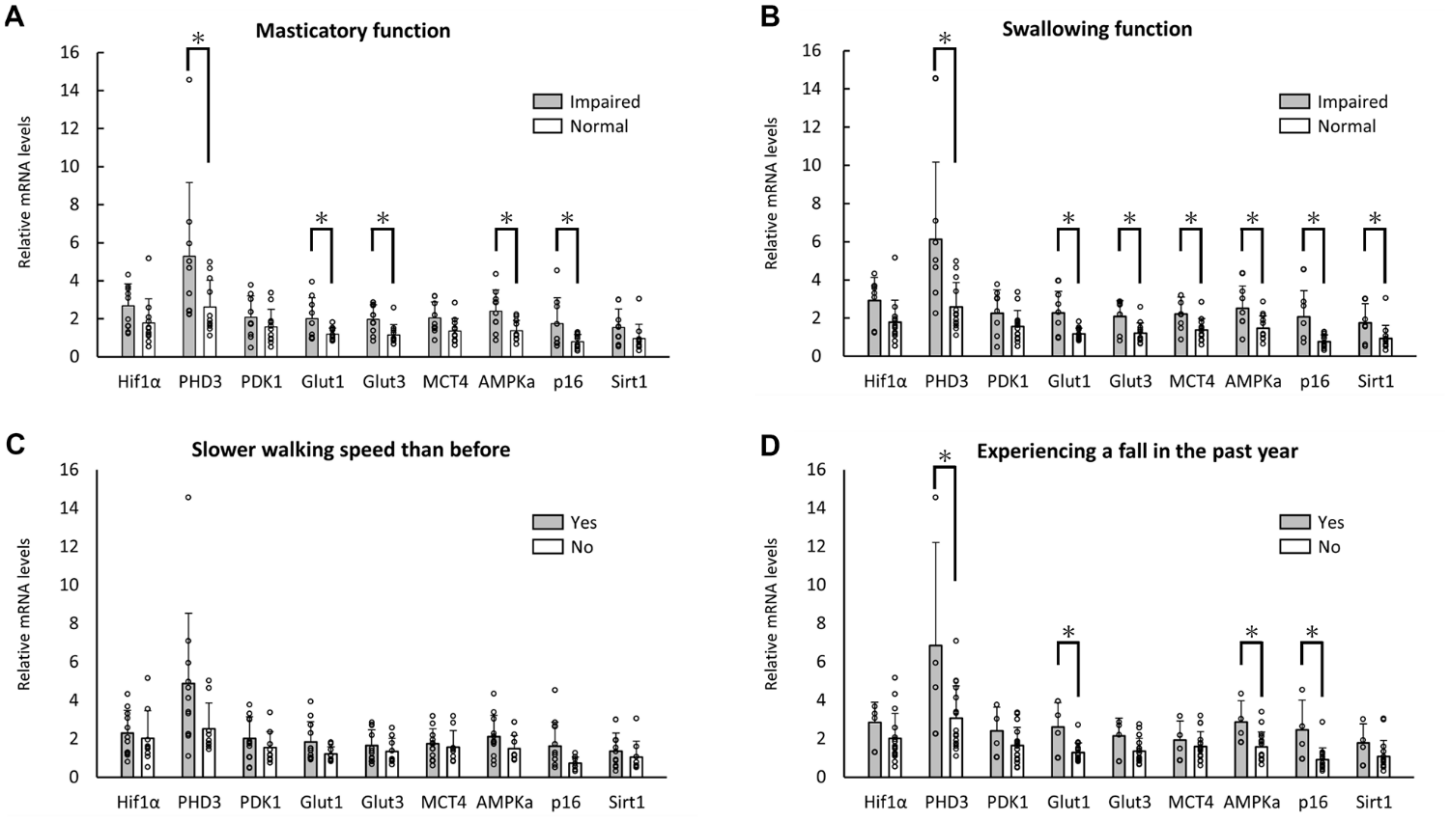
**Changes in RNA expression with oral and physical functions.** Significant differences in RNA expression levels between aged individuals with and without symptoms of masticatory disturbance (**A**) or swallowing dysfunction (**B**). No significant differences were observed in slower walking speed (**C**), but significant differences were observed in the experience of a fall in the past year (**D**). *p<0.05 (**A**, **B**, **D**).

Nine of the 20 enrolled aged individuals reported symptoms of masticatory disturbance in the health assessment questionnaire on physical frailty. Figure 1A shows the results of quantitative PCR (qPCR) analysis of the RNA expression levels of metabolism-related genes in each group. Significant differences were observed in the levels of metabolism-related genes, including PHD3, Glut1, Glut3, AMPKa, and p16. Seven of the 20 enrolled individuals reported symptoms of swallowing dysfunction. A significant difference was seen in the expression levels of PHD3, Glut1, Glut3, MCT4, AMPKa, p16, and Sirtuin1 (Sirt1) (Figure 1B). Eleven of the 20 enrolled individuals reported slower walking speed than before. No significant difference was observed in the expression levels of WBC (Figure 1C). Four of the 20 enrolled individuals reported experiencing a fall in the past year. A significant difference was observed in the expression levels of metabolism-related genes, including PHD3, Glut 1, AMPKa, and p16 (Figure 1D).

### Changes in WBC RNA expression profiles in elderly with body weight loss and weekly exercise

**Figure 2:**
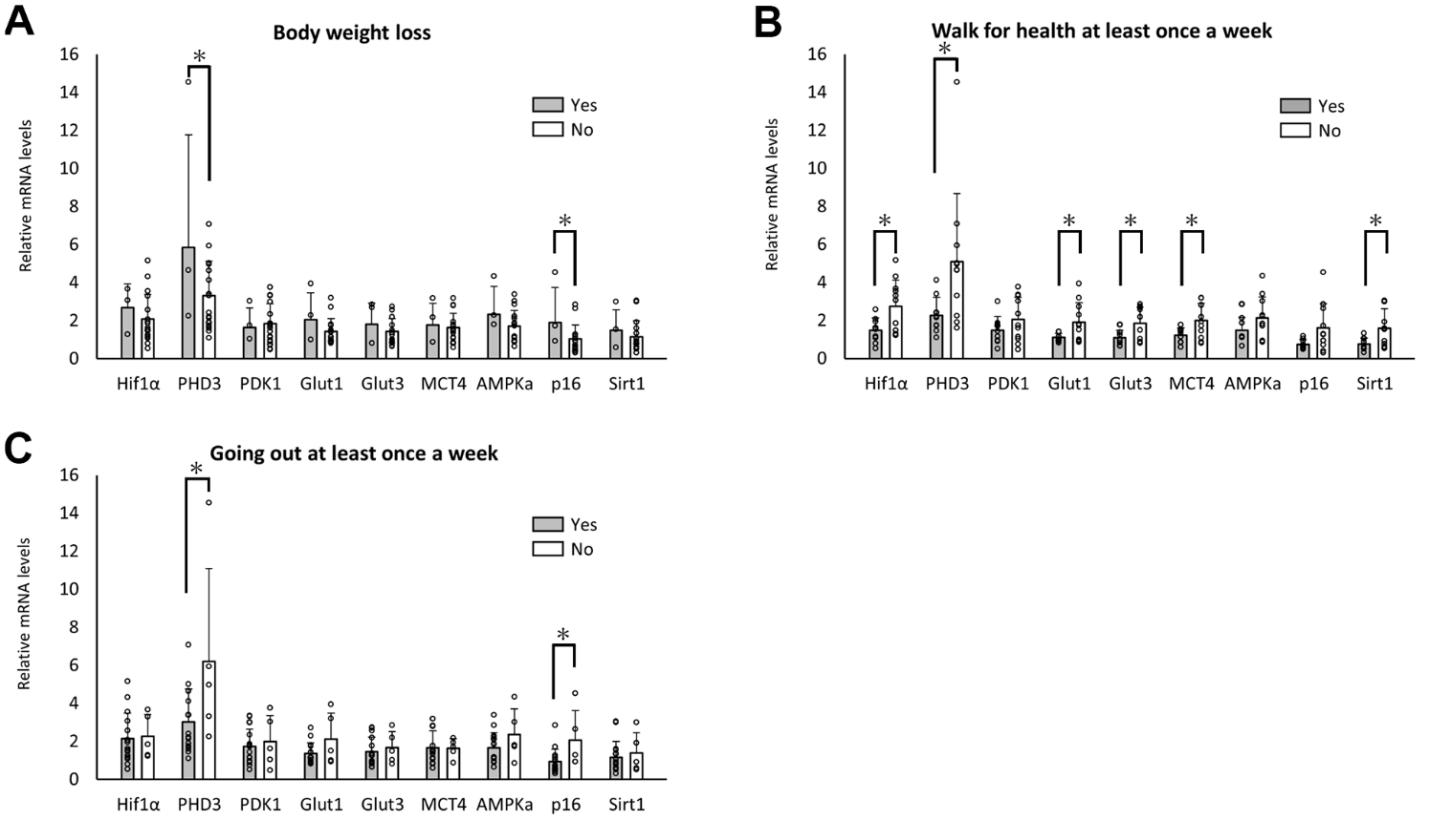
**Changes in RNA expression with body weight loss and weekly exercise.** Significant differences in RNA expression levels between aged individuals with and without body weight loss (**A**), walking habits (**B**), and going out at least once per week (**C**). *p<0.05 (**A**–**C**).

Three of the 20 enrolled individuals reported body weight loss (>2 kg) in the past 6 months. A significant difference was observed in the expression levels of PHD3 and p16 (Figure 2A). Nine of the 20 enrolled individuals reported healthy walking habits at least once a week. A significant difference was observed in the expression levels of metabolism-related genes, including Hif1α, PHD3, Glut 1, Glut3, MCT4, and Sirt1, (Figure 2B). Fifteen of the 20 enrolled individuals reported going out at least once a week. A significant difference was seen in the expression levels of PHD3 and p16 (Figure 2C).

### Changes in WBC RNA expression profiles in elderly with cognitive impairment

**Figure 3:**
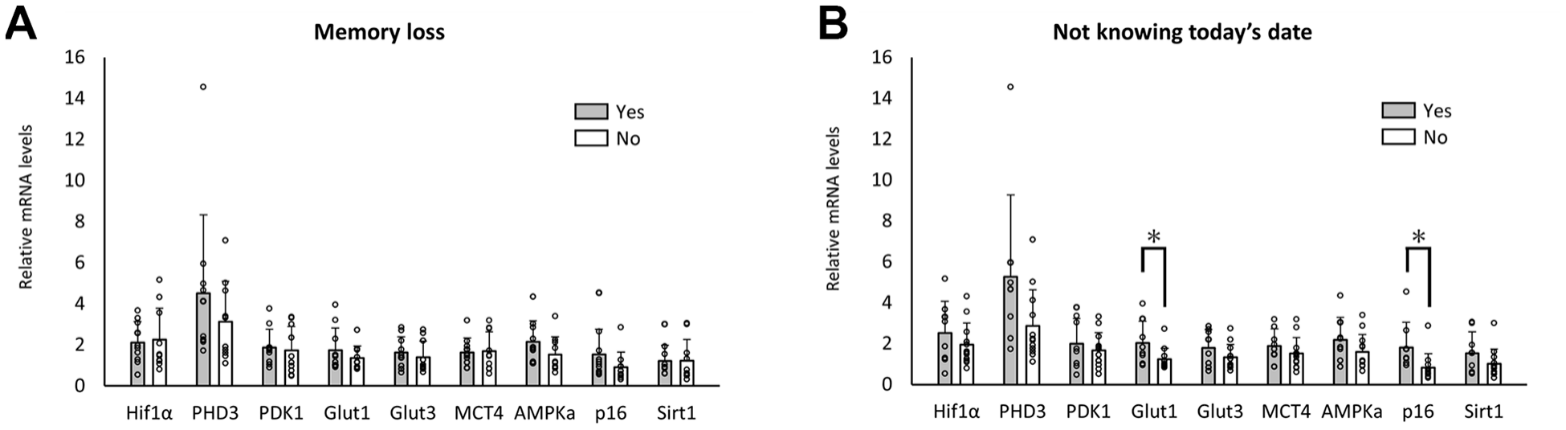
**Changes in RNA expression pattern in elderly with cognitive impairment.** No significant difference in the level of RNA expression related to memory loss (**A**). A significant difference in RNA expression levels was observed when reporting knowledge of today’s date (**B**). *p<0.05 (**B**).

Ten of the 20 enrolled individuals reported experiencing memory loss, as pointed out by a family member or friend. As shown in Figure 3A, no difference was observed in RNA expression between the groups with and without such experiences. Eight of the 20 enrolled individuals reported not knowing the today’s date. A significant difference in the RNA expression of Glut1 and p16 was observed (Figure 3B).

### Change in WBC RNA expression profiles with the other reported inquiries

**Figure 4:**
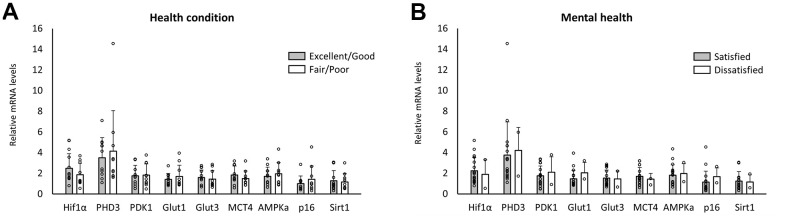
**Changes in RNA expression pattern with the other inquiries.** No significant differences were seen in the level of RNA expression between the elderly with and without reporting health conditions as excellent or good (**A**) and satisfied or moderately satisfied with daily life (**B**).

Ten of the 20 enrolled individuals reported that their health condition was excellent or good. Seventeen enrolled individuals reported being satisfied or moderately satisfied with their daily lives. RNA analysis of circulating WBC revealed no differences in RNA expression patterns between the groups (Figure 4A, 4B). Nineteen of the twenty enrolled individuals selected the same answers to the remaining questions, and no statistical analyses were performed.

### Correlation between weekly exercise and physical frailty

**Figure 5:**
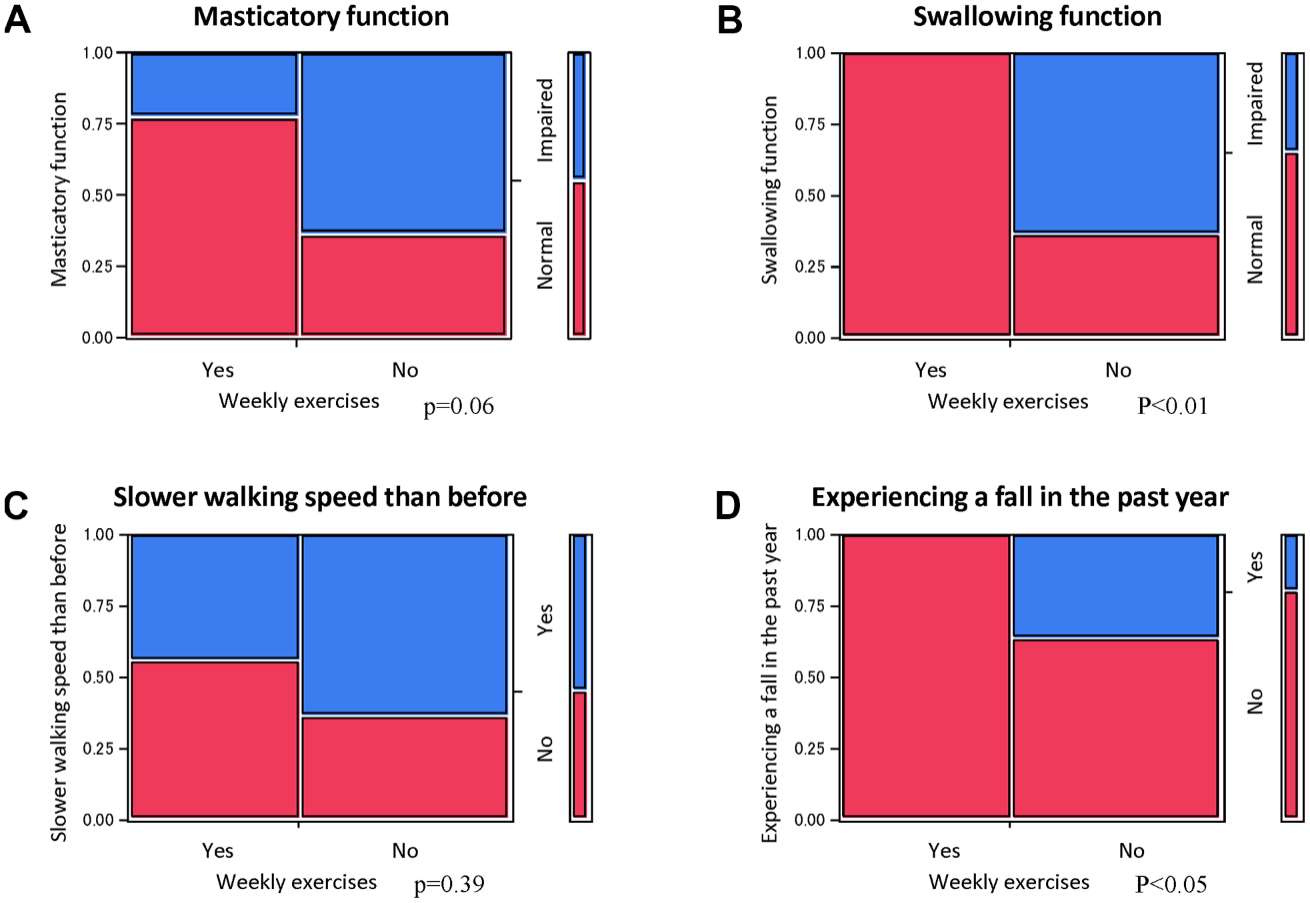
**Correlation between weekly exercise and physical frailty.** The results of the chi-square test for the correlation between weekly exercise and masticatory disturbance (**A**), swallowing dysfunction (**B**), slower walking speed (**C**), and falling in the past year (**D**). Aged individuals who exercised weekly showed significantly better swallowing function and less falling.

A correlation was observed between walking for health at least once a week and physical frailty (Figure 5A–5D). Aged individuals who participated in weekly exercise exhibited significantly better swallowing function and less falling.

### Impact of age on oral/physical function and RNA expression in WBC

**Figure 6:**
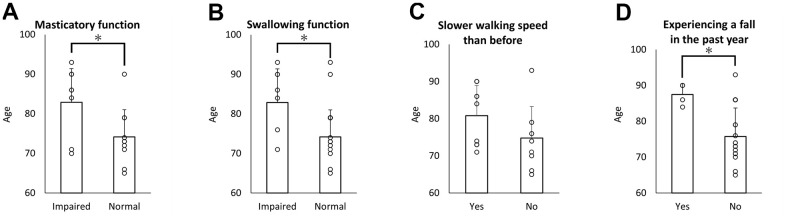
**Oral/physical dysfunction and age.** The mean age of elderly with masticatory disturbances (**A**) and swallowing dysfunction (**B**) was higher than that of elderly without each symptom. No significant difference in the mean age was observed between elderly who reported or did not report slower walking speed (**C**). The mean age of the elderly who experienced a fall in the past year was higher than that of the elderly without a fall (**D**). *p<0.05 (**A**, **B**, **D**).

**Figure 7:**
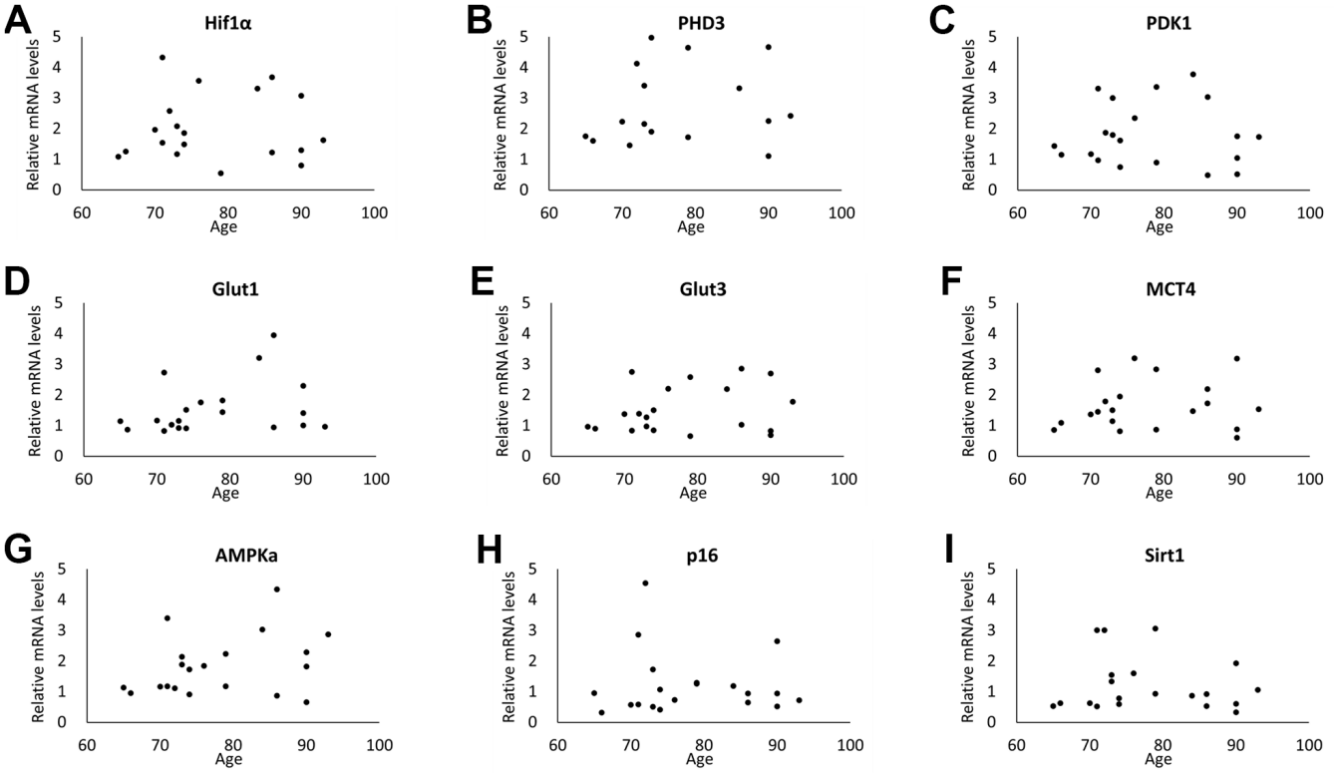
**Correlation between age and RNA expressions in WBC.** No significant correlation was observed in RNA expression of Hif1α (**A**), PHD3 (**B**), PDK1 (**C**), Glut1 (**D**), Glut3 (**E**), MCT4 (**F**), AMPKa (**G**), Sirt1 (**H**) and p16 (**I**). (n=20 each).

The distribution of age with or without oral or physical dysfunction is shown in Figure 6A–6D. The enrolled individuals with masticatory disturbance (Figure 6A) and swallowing dysfunction (Figure 6B) showed higher age, compared to individuals without dysfunction. No significant difference in age was observed between individuals with and without slower walking speeds (Figure 6C). The enrolled individuals who had experienced a fall in the past year were older than those without the experience (Figure 6D). Figure 7A–7I shows the correlation between age and RNA expression in WBC. No significant correlation was observed.

### Cellular interaction between circulating WBC and cells in muscle tissue of mice

**Figure 8:**
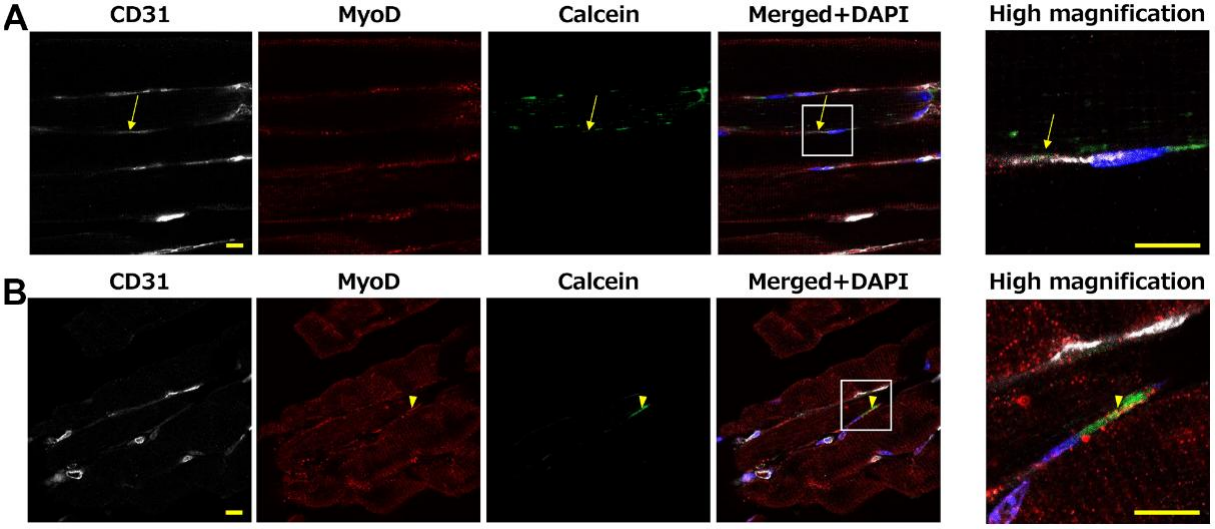
**Mouse muscle at 10 minutes after Calcein loaded WBC intravenous injection.** Calcein signals were observed in the muscle tissue, including CD31-positive endothelial cells (**A**: arrow) and MyoD-positive satellite cells (**B**: arrowhead). High-magnification images of the white squares are shown in right panel. Scale bar = 10 μm (**A**, **B**.; left and right panels).

Multiple causes of physical frailty have been proposed including malnutrition, hormonal changes, impaired capillary blood flow, and satellite cells [10]. Endothelial cells are prominent cells that regulate capillary blood flow, and satellite cells are skeletal muscle stem cells that play a significant role in the maintenance, repair, and regeneration of muscle fibers. Based on these findings, we investigated the association between circulating WBC and endothelium/satellite cells in mice. The water-soluble green fluorescent molecule, Calcein, was loaded into WBC, the WBC were injected via tail vein, and the transfer of Calcein into endothelium/satellite cells was investigated at 10 minutes after WBC injection. Calcein-positive signals were observed in the muscle tissue, including the endothelium and satellite cells (Figure 8A, 8B). The mean ratio of Calcein-positive endothelium or satellite cells in total endothelium or satellite cells was 5.4±5.7% or 6.7±9.4%, respectively. These findings indicate that a direct link between circulating WBC and cells in muscle tissues is not rare phenomenon. No green fluorescence-positive endothelium or satellite cells were observed in the mice that received PBS (data not shown).

## DISCUSSION

In this explorative analysis, we found that increased RNA expression of metabolism-related genes in circulating WBC was significantly correlated with impairment of oral and physical functions in the elderly. Furthermore, decreased RNA expression of metabolism-related genes in WBC was observed in the elderly with walking habits compared to those without the habits. Our results suggest the level of metabolism-related RNA expression in circulating WBC can be a marker of physical frailty, as well as the level of exercise in the elderly.

**Figure 9:**
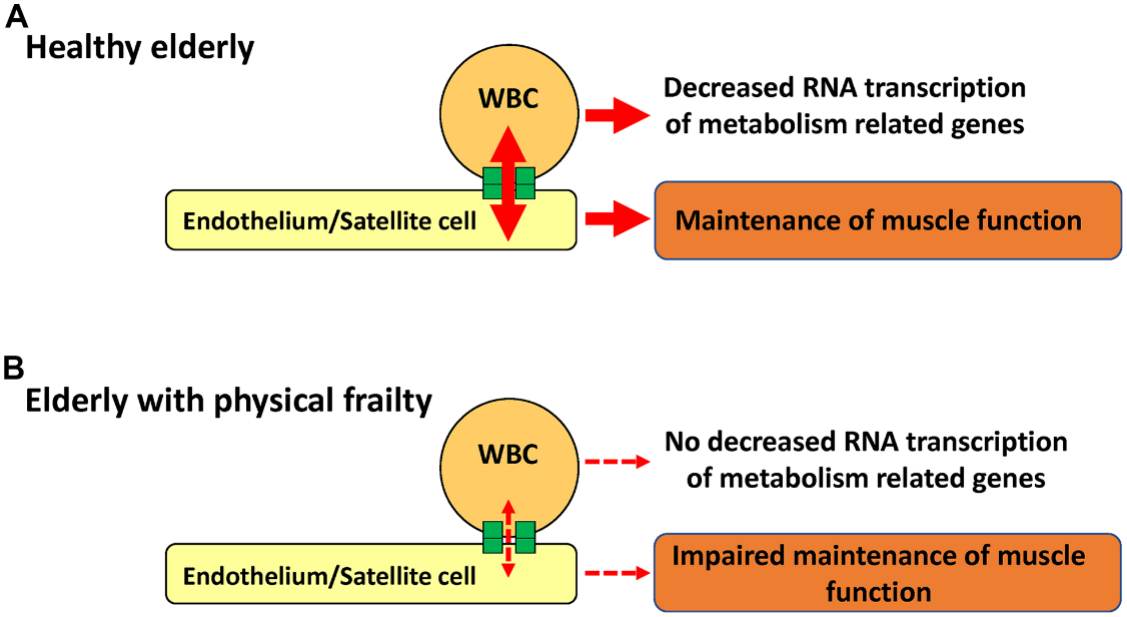
**Schematic illustration of our hypothesis that links RNA transcription in WBC and physical frailty.** Direct cell-cell interaction between circulating WBC and endothelium/satellite cells in healthy aged individuals decreases RNA transcription of metabolic-related genes in WBC while maintaining muscle function (**A**). In contrast, impaired cell-cell interactions result in no changes in the RNA transcription of metabolic-related genes in WBC, with impaired maintenance of muscle function (**B**).

Gap junction has a significant impact on the metabolic status of connected cells [8] and play an essential role in development, cellular differentiation, and regeneration [5, 9, 11]. Transplantation of hematopoietic stem cells in animal models of stroke, aged dementia, and bone fracture have been shown to activate endothelial cell and tissue stem cell via gap junction [4, 9, 12]. Similar to hematopoietic stem cells, *in vivo* analysis in mice has revealed that WBC provide water-soluble small molecules to the endothelium and neuronal stem cells in the brain [4]. In addition, *in vitro* analysis revealed that the expression of metabolism-related RNA in WBC is reduced by cell-cell interactions with endothelial cells via gap junction [3]. In this article, we report that WBC provide water-soluble small molecules to the endothelium and satellite cells in the muscle using an animal model. These findings indicate that the cellular interaction among circulating WBC, endothelial cells, and tissue stem cells, as well as potentially others, is a general physiological phenomenon that contributes to the maintenance and activation of tissues and organs, resulting in decreased RNA transcription of metabolism-related genes in circulating WBC (Figure 9). These findings indicate a previously unknown physiological role for circulating WBC, in addition to immune response and inflammation control, and explain the rejuvenation of progenitor cells in aged mice by exposure to a young systemic environment via heterochronic parabiosis [13].

The major causes of physical frailty in the elderly are impaired metabolism, reduced capillary blood flow, decreased number of muscle satellite cells, and lack of exercise [14]. Recently, impaired metabolism, reduced capillary blood flow, a decreased number of neuronal stem cells, and lack of exercise had been proposed to be the major cause of Alzheimer’s disease in the elderly [14–16]. The level of gap junction in the endothelium is downregulated with aging [17] and the blockade of cellular interactions between endothelial cells and WBC via gap junction has been shown to increase the RNA transcription of glycolysis-related genes in WBC [3]. Our findings show that increased RNA expression of metabolism-related genes in circulating WBC was significantly correlated with impairment of oral and physical function in the elderly, likely due to a decrease in cellular interaction via gap junction. From a therapeutic perspective, molecules that activate cell-cell interactions via gap junction, such as histone deacetylase, have been proposed as candidates for novel therapies [18]. Interactions via gap junction can be activated by the increased transcription of gap junction genes, reduced degradation of gap junction proteins, and activated channel opening [5]. Pharmacological agents or dietary supplements that can activate cell-cell interactions via gap junction may also be novel treatments for age-related diseases, including physical frailty and Alzheimer’s disease.

**Figure 10:**
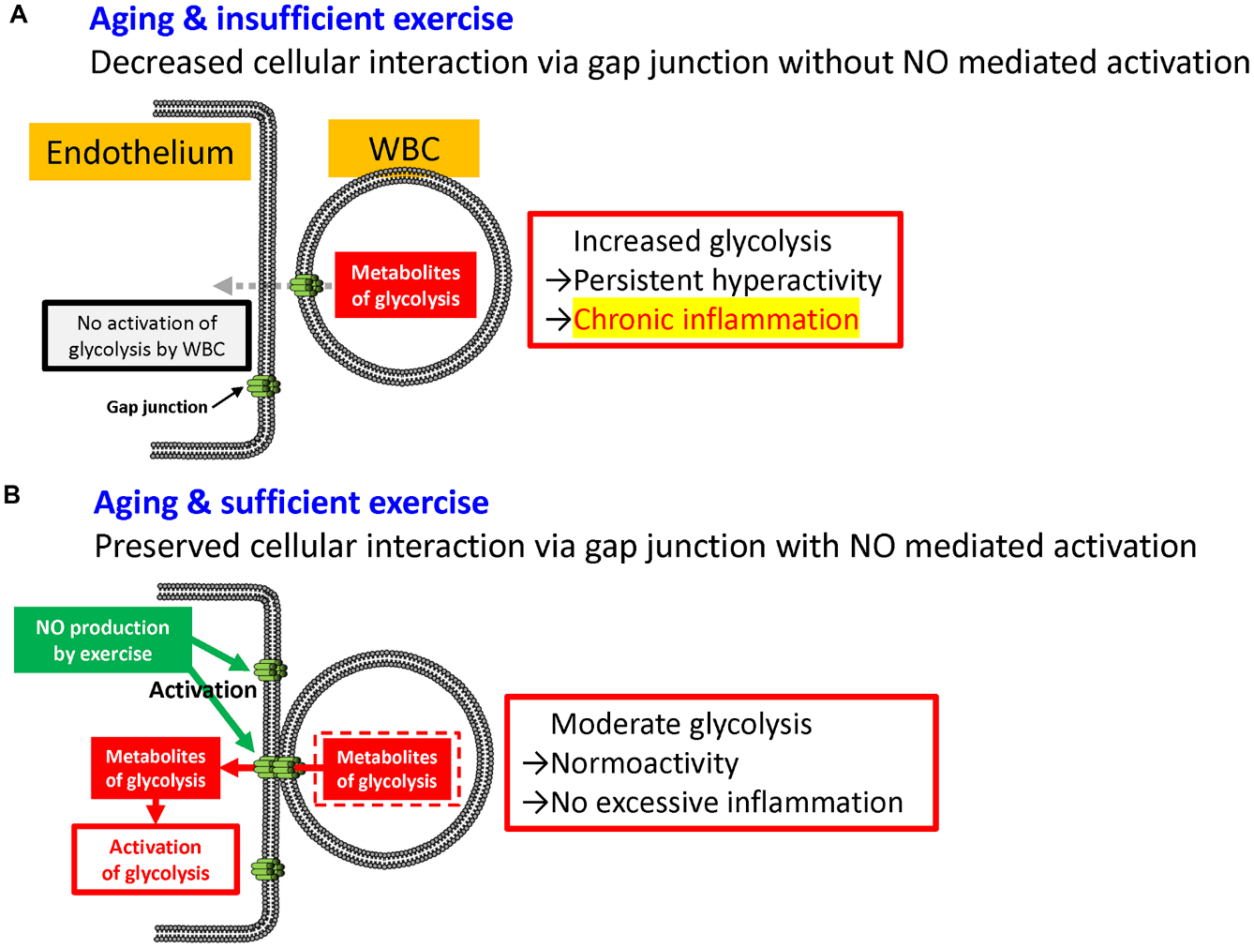
**Possible mechanism linking the metabolism of leukocyte and chronic inflammation.** Without (**A**) and with (**B**) sufficient exercise. Gap junction-mediated cellular interactions decreased with aging which results in the increased glycolysis in WBC with hyperactivity. In contrast, exercise is known to activate *de novo* gap junction-mediated interaction that results in moderate glycolysis in WBC.

In this article, we report that weekly exercise reduces the RNA expression of glycolysis-related genes in WBC. Exercise augments endothelial nitric oxide (NO)-dependent vasodilatation in both small and large vessels [19] and NO enhances *de novo* gap junction formation in endothelial cells [20]. These findings also indicate that weekly exercise promotes gap junction formation between endothelial cells and WBC via activation of NO production, resulting in decreased RNA expression of glycolysis-related genes in WBC. Our results will enable the evaluation of the effect and adequacy of exercise as a surrogate marker, and will be useful for setting appropriate exercise intensity in the elderly. Further clinical studies are necessary to clarify the link between appropriate exercise, NO production, and RNA expression and its effect on physical frailty and Alzheimer’s disease. Chronic inflammation with aging is one of the major causes of physical frailty and Alzheimer’s disease in elderly [21, 22]. Exercise is one of the best interventions to prevent physical frailty [23] and dementia [24] that activates satellite cells [25] and neuronal stem cells [26], respectively. Furthermore, exercise has the potential to moderate inflammation [27] and increased glycolysis in WBC that enhances inflammation [28]. Although various factors have been proposed as possible causes of chronic inflammation with aging [21, 29, 30], our results suggest that the link among insufficient exercise, physical frailty, and increased glycolysis in WBC is a novel aspect of chronic inflammation with aging (Figure 10).

The enrolled elderly with masticatory disturbance, swallowing dysfunction, and experiencing a fall in the past year showed higher age, compared to elderly without each dysfunction. These results are consistent with the pathogeny that aging is the most significant factor associated with physical frailty. In contrast, no correlation was observed between age and RNA expression levels. These findings indicate that increased expression of metabolism-related RNA in aged individual with symptoms of physical frailty is not simply due to aging but may reflect the pathology that causes physical frailty; however, further studies with multivariate statistical analyses including a significant number of aged individuals are required to confirm this. Hif1α is one of the master regulators of cellular metabolism that regulates the expression of energy source transporters [31] and PHD3 is known to be a down-stream gene of Hif1α [32]. As the activity of Hif1α is regulated by its disassembly by PHD3 in an oxidant dependent manner [33], a discrepancy between RNA expression of Hif1α and PHD3 would be explained by the change of degradation rate of Hif1α rather than its RNA transcription.

Our findings reported from this study have limitations, as this was largely an explorative study. The major focus of a related clinical study was to explore the reproducibility of the results in aged mice [3]. Therefore, only a small number of aged individuals were enrolled without a comprehensive objective examination, and the number was insufficient for multivariate analysis. In addition, the evaluated RNAs were limited to metabolism-related genes based on the results obtained in aged mice [3], and the mechanism linking RNA expression in WBC and physical frailty via gap junction has not been elucidated. Further confirmatory studies with increased numbers, including females, and measurements of other parameters are necessary as the next step. The impact of gap junction is widespread in a variety of physiological and pathological processes [34] and gap junction channels are regulated by complex mechanisms [35]. One of the universal functions of gap junction is metabolic cooperation between cells [36] and cell-cell interactions between endothelial cells and WBC have been shown to reduce the RNA expression of metabolism-related genes in WBC [3]. Further basic research and clinical studies are required to reveal the full role of gap junction-mediated cell-cell interactions in aging and their impact on the onset of physical frailty.

In conclusion, our results indicate that the increased RNA expression of metabolism-related genes in circulating WBC can serve as a marker of physical frailty in aged humans, which is consistent with the results obtained in aged mice. Our findings suggest a previously unknown physiological role for circulating WBC and reveal a novel aspect of chronic inflammation with aging.

## MATERIALS AND METHODS

### Enrollment in clinical studies

The protocol in this study was designed based on results obtained in aged male mice [3], and the primary focus of this study was to explore the similarities and differences between aged humans and mice. Twenty elderly volunteers (minimum age, 65 years), all of whom provided written informed consent, were enrolled in this study. Only males were enrolled to avoid possible variations between the sexes. The link between RNA expression profiles of metabolism-related genes in circulating WBC and the limited number of reports related to frailty [3] was investigated to minimize the amount of blood collected. The new Health Assessment Questionnaire for the National Screening Program for Older Adults in Japan [2] was used to evaluate frailty symptoms. The exclusion criteria, list of questionnaires, and answers of all enrolled aged individuals are shown in Tables 1, 2 and Supplementary Table 1, respectively.

**Table 1:** Exclusion criteria.

1	Diabetes Mellitus
2	During cancer treatment or less than 5 years after the end of treatment
3	Thyroid disease
4	Infectious diseases (hepatitis B, hepatitis C, AIDS)
5	Fever at the day of blood collection
6	Chronic subdural hematoma
7	Hydrocephalus (normal pressure)
8	During hemodialysis
9	COPD (Chronic Obstructive Pulmonary Disease)
10	During home oxygen therapy
11	Smoking in the last 12 months
12	Taking drugs that have an effect on the immune system (anti-cancer drugs, steroids, antirheumatic drugs)
13	Serious complications, or have a history of these and judged to be ineligible for this study
14	Judged by the principal investigator to be ineligible

**Table 2:** The health assessment questionnaire for frailty of elderly.

**Domain**	**Item**
Health condition	How is your health condition?
Mental health	Are you satisfied with your daily life?
Eating behavior	Do you eat three meals a day?
Oral function	1. Do you have any difficulties eating tough foods when compared to 6 months ago?
	2. Have you choked on your tea or soup recently?
Bodyweight loss	Have you lost 2 kg or more in the past 6 months?
Physical function and falls	1. Do you think you walk slower than before?
	2. Have you experienced a fall in the past year?
	3. Do you go for a walk for your health at least once a week?
Cognitive function	1. Do your family or friends point out your memory loss?
	2. Do you find yourself not knowing today’s date?
Smoking	Do you smoke?
Social participation and support	1. Do you go out at least once a week?
	2. Do you maintain regular communications with your family and friends?
	3. When you feel ill, do you have anyone to reach out/talk to?

### Quantitative PCR (qPCR) analysis of circulating leukocyte

Fasting blood samples were obtained from the median cubital vein, and RNA expression in WBC was evaluated. WBC RNA was stabilized using a PAXgene Blood RNA Tube (#762165, BD Bioscience, NJ, USA). Total RNA was isolated using NucleoSpin RNA (Takara, Kyoto, Japan) according to the manufacturer’s protocol. cDNA was synthesized from 0.3 μg total RNA using PrimeScript™ II 1st strand cDNA Synthesis Kit (Takara). Transcription of mRNA was analyzed by PowerUp™ SYBR™ Green Master Mix (Applied Biosystems, CA, USA) and the AriaMx real time quantitative PCR System (Agilent, CA, USA). 18S RNA was used as the reference gene. A list of target genes, primer sequences, and amplification protocols is shown in Table 3.

**Table 3:** Target genes, primer list and amplification protocol.

**Gene**	**NCBI accession No.**		**Sequence**
hHif1α	NM_001530.4	Forward	CCAGACGATCATGCAGCTACT
(Hypoxia inducible factor 1α)		Reverse	TGATTGCCCCAGCAGTCTAC
hPHD3	NM_022073.3	Forward	GATCGTAGGAACCCACACGA
(Prolyl hydroxylase 3)		Reverse	TCAGAGCACGGTCAGTCTTC
hPDK1	NM_001278549.1	Forward	GCAAAATCACCAGGACAGCC
(Pyruvate dehydrogenase kinase 1)		Reverse	TCTGTTGGCATGGTGTTCCA
hGlut1	NM_006516.3	Forward	CCTGCAGTTTGGCTACAACAC
(Glucose transporter 1)		Reverse	CAGGATGCTCTCCCCATAGC
hGlut3	NM_006931.3	Forward	ATTACAGCGATGGGGACACA
(Glucose transporter 3)		Reverse	GCCAAATTGGAAAGAGCCGA
hMCT4	NM_001042423.3	Forward	CGGAGCATCATCCAGGTCTAC
(Monocarboxylate transporter 4)		Reverse	GGCTGGAAGTTGAGTGCCAA
hAMPKa	NM_001355034.2	Forward	CGGCAAAGTGAAGGTTGGC
(AMP-activated protein kinase A)		Reverse	CCTACCACATCAAGGCTCCG
p16	NM_001195132.2	Forward	CTTCCTGGACACGCTGGTG
(cyclin-dependent kinase inhibitor 2A)		Reverse	GCATGGTTACTGCCTCTGGTG
Sirt1	NM_001142498.2	Forward	TAGACACGCTGGAACAGGTTGC
(sirtuin 1)		Reverse	CTCCTCGTACAGCTTCACAGTC
h18s	NR_003286.4	Forward	GGCCCTGTAATTGGAATGAGTC
(18s ribosomal RNA)		Reverse	CCAAGATCCAACTACGAGCTT
**Amplification protocol**			
**Segment**	**Plateau**	**Temperature**	**Duration**
Hot Start	1	50	0:03:00
Hot Start 2	1	95	0:03:00
Amplification	1	95	0:00:05
Amplification	2	60	0:00:30
Melt	1	95	0:00:30
Melt	2	65	0:00:30
Melt	3	95	0:00:30

### Cell transplantation into mice

After deep anesthesia with isoflurane (WAKO, Osaka, Japan), mouse peripheral blood was obtained by heart puncture from 6-week-old male CB-17 mice (CLEA Japan, Tokyo, Japan), and mononuclear cells were isolated by Ficoll-Paque (Cytiva, Washington DC, USA) density-gradient centrifugation, as described previously [9]. Cells were incubated with 5 μM Calcein acetoxymethyl ester (Calcein-AM; Dojindo, Kumamoto, Japan) for 30 minutes at 37° C. Calcein-AM is a non-fluorescent cell-permeable dye that is converted to green-fluorescent Calcein in live cells after acetoxymethyl ester hydrolysis by intracellular esterases. The molecular weight of Calcein is 623, and it is a non-cell-permeant molecule [37]. Calcein in the cytoplasm of transplanted cells is known to be transferred to other recipient cells via gap junction in 10 minutes after intravascular transplantation [9, 11]. Calcein-loaded cells were washed twice with PBS before transplantation. These cells (1×10^6^ cells in 10 μL PBS) were then injected via the tail vein. Mouse thigh muscles were removed by cervical dislocation 10 minutes after cell transplantation. The muscle was fixed with 2% paraformaldehyde and cut into sections (20 μm) using a cryostat (Leica, Wetzlar, Germany). The sections were immunostained with antibodies against the endothelium (anti-CD31 antibody, BD Biosciences, USA) and satellite cells (anti-MyoD antibody, Bioss, UK). Goat anti-Rat IgG Alexa Fluor 647 and Goat anti-Mouse IgG Alexa Fluor 555 were used as the secondary antibody for anti-CD31 and anti-MyoD antibodies, respectively. A confocal microscope (LSM990; Carl Zeiss, Oberkochen, Germany) was used to collect images for further analysis. Three mice were injected with Calcein-loaded cells. Representative images are presented in the Results section. The absence of false-positive Calcein signals was confirmed in the three mice without cell injection. All the mice in each group exhibited similar results. No mice were excluded from the study. CD31, Calcein or MyoD, and Calcein double-positive cells in the muscle were considered endothelial cells or satellite cells that received Calcein from the injected mononuclear cells via gap junction. The ratio of Calcein and CD31-positive cells or Calcein and MyoD-positive cells to total CD31- or MyoD-positive cells was counted by blinded investigators (n=6 fields each [n=2 fields for each mouse, n=3 mice in each group]). Seven mice were used, including one used for cell harvesting. No randomization was performed.

### Data analysis

Normal distribution of the data was confirmed using JMP 7.0 (JMP Statistical, NC, USA) with the Shapiro-Wilk test. The chi-square test was used for nonparametric statistics. Individual comparisons were performed using the Student’s t-test. The correlation between the RNA transcription of metabolism-related genes in circulating WBC and age was evaluated by linear regression analysis. In all experiments, the mean ± SD are reported.

### Data availability statement

The datasets used and/or analyzed in the current study are available from the corresponding author upon reasonable request.

## Supplementary Material

Supplementary Table 1
